# The effects of optically and digitally simulated aniseikonia on stereopsis

**DOI:** 10.1111/opo.12973

**Published:** 2022-03-06

**Authors:** David A Atchison, Thien Nguyen, Katrina L Schmid, Archayeeta Rakshit, Alex S Baldwin, Robert F Hess

**Affiliations:** ^1^ Centre for Vision and Eye Research Queensland University of Technology Kelvin Grove Queensland Australia; ^2^ McGill Vision Research Unit Department of Ophthalmology & Visual Sciences McGill University Montreal Quebec Canada

**Keywords:** aniseikonia, anisometropia, meridional, stereopsis

## Abstract

**Purpose:**

To simulate both lens‐induced and screen‐induced aniseikonia, and to assess its influence on stereopsis. Additionally, to determine if screen‐based size differences could neutralise the effects of lens‐induced aniseikonia.

**Method:**

A four‐circle (4‐C) paradigm was developed, where one circle appears in front or behind the others because of crossed or uncrossed disparity. This stereotest was used for three investigations: (1) Comparison with the McGill modified random dot stereogram (RDS), with anisometropia introduced with +2 D spheres and cylinders, and with aniseikonia introduced with 6% overall and 6% meridional (×180, ×90) magnifiers before the right eye; (2) Comparison of lens‐induced and screen‐induced 6% overall and meridional magnifications and (3) Determining if lens and screen effects neutralised, by opposing 6% lens‐induced magnification to the right eye with screen‐inducements of either 6% left eye magnification or 6% right eye minification. A pilot study of the effect of masking versus not masking the surround was also conducted.

**Results:**

The 4‐C test gave higher stereo‐thresholds than the RDS test by 0.5 ± 0.2 log units across both anisometropic and aniseikonic conditions. However, variations in power, meridian and magnification affected the two tests similarly. The pilot study indicated that surround masking improved neutralisation of screen and lens effects. With masking, lens‐induced and screen‐induced magnifications increased stereo‐thresholds similarly. With lens and screen effects opposed, for most participants stereo‐thresholds returned to baseline for overall and ×180 magnifications, but not for ×90 magnification. Only three of seven participants showed good compensation for ×90 magnification.

**Conclusions:**

Effects of lens‐induced aniseikonia on stereopsis cannot always be successfully simulated with a screen‐based method. The ability to neutralise refractive aniseikonia using a computer‐based method, which is the basis of digital clinical measurement, was reasonably successful for overall and ×180 meridional aniseikonia, but not very successful for ×90 aniseikonia.

**Key Points**

- The effects of lens‐induced aniseikonia on stereopsis can be simulated by a screen‐based method.
- The neutralisation of optical aniseikonia by screen‐induced aniseikonia was better for overall and ×180 magnifications than for ×90 magnifications.

## INTRODUCTION

Binocular vision provides primates with several important advantages beyond the small (approximately 40%) improvement in visual acuity. It provides better postural stability,^1^ reading performance,^2^ driving performance,^3^ depth perception,^4^ fine motion control^5^, ^6^, ^7^, ^8^ and sporting performance.^9^ Anisometropia of 2D or more, which occurs in 1.7% of the population,^10^ is an impediment to binocular vision for the following reasons: uncorrected anisometropia introduces uniocular blur which reduces stereoscopic ability; corrected anisometropia with spectacle lenses introduces aniseikonia, which can also detrimentally affect stereopsis and fusion and finally, anisometropia in early life can result in a more long‐term visual deficit, amblyopia, which can also reduce stereopsis due to reduced visual acuity and suppression of the amblyopic eye. Understanding the potential impact of anisometropia on binocular function, particularly stereopsis, requires an appreciation of how blur and aniseikonia individually impact stereoscopic ability, as well as an evaluation of the adequacy of the present methods for aniseikonia measurement.

In previous studies, the first issue was addressed by investigating the effects of blur (lens‐induced monocular and binocular) and of aniseikonia on stereopsis.^11^, ^12^ Trial lenses of differing power or magnification produced blur or aniseikonia, with participants making stereoscopic judgements on a computer monitor using a standard random dot stereotest that determined the stereoacuity threshold. Stereothresholds were influenced by the meridian of blur but not by the meridian of aniseikonia, and for binocular blur conditions stereothresholds were more affected when blur meridians were orthogonal than when they were parallel in the two eyes.

Concerning the second issue, aniseikonia can be simulated by the spatial scaling of stimuli with lenses (optical method) or on a monitor (digitally‐induced screen‐based method). Accordingly, the purpose of this study was to determine whether different sized stimuli provided to the right and left eyes, produced digitally on a computer screen, give similar stereo‐thresholds to having different magnification lenses before each eye. This information is important for assessing the adequacy of present digital methods for measuring optical‐induced aniseikonia, all of which use stimulus scaling.^13^

Recalculating the size of the stimulus images on a monitor would be problematic with a random dot‐based stereopsis test. For this reason, a figural‐based test was developed that comprised four circles (4‐C test) presented in a diamond arrangement: one to the right, one to the left, and one up and one down from the screen centre. These circle stimuli are generated simply as functions of the *x*, *y* coordinates on the display. These coordinates can easily be transformed to produce the required distortions. These distortions also affected the crossed or uncrossed disparity between the right and left eye targets which was applied to one of the circles. Furthermore, we expect that the effects of aniseikonia will be less strong for the stimuli formed of four discrete circles compared to the random dot stimulus. We anticipate one of the consequences of aniseikonia for the random dot stimuli is that it will result in mismatched correspondence. That is to say that two dots which would not match up with each other before the distortion will be erroneously matched after the distortion. For the 4‐C test, the stimuli are formed of four discrete circles. Therefore, we expect there to be less chance of a mismatched correspondence between the images.

We hypothesised that interocular difference in target size would be similar to inducing aniseikonia with size lenses. This is the basis for clinical measurement of aniseikonia using digital devices such as the Awaya Aniseikonia Test (OCULUS, oculus.de.en) and the Aniseikonia Inspector.^14^ To determine if this was indeed the case, three investigations were performed. Firstly, we compared the new 4‐C stereo test against the random dot stereogram test used in previous studies. Then we compared the effects of optical aniseikonia introduced by lenses against a simulation performed by physically changing the images on the screen. Finally, we set out to determine whether equal and opposite lens‐induced and screen size differences could negate each other.

## METHODS

### Participants

Participants were recruited from the student and staff population of the Queensland University of Technology (QUT). Screening was undertaken to ensure all participants had good baseline habitual visual acuities (better than logMAR 0.0 (6/6)) and normal stereoacuity thresholds (≤40 sec arc) when wearing their correction. The study complied with the tenets of the Declaration of Helsinki, approval was obtained from the QUT University Human Research Ethics Committee, and informed written consent was obtained from all participants after procedures were explained.

There were 14 participants aged 19–65 years, with good general and ocular health including normal binocular vision. Mean spherical refractions ranged from +0.37 D to –6.75 D. Participants had ≤0.75 D differences in the meridional refraction components of their two eyes. Eight participants were corrected with spectacles, trial lenses or contact lenses. Two presbyopic participants were given a near add of +1 D to correct vision for the test distance. Ten participants took part in Experiment 1, two took part in pilot Experiment 2, and seven took part in Experiment 3 (two in common with Experiment 1).

### Stereogram tests

Stimuli were displayed on a 24‐inch ASUS VG248QE 3D monitor (ASUS, asus.com) and viewed through synchronised shutter glasses (3D vision 2 ‐ model P1431, NVIDIA, nvidia.com/en‐us) worn over the spectacle correction and magnifying lenses. Room lights were turned off. Two tests were used to determine stereoacuity. The first test was the McGill modified random dot stereogram used in the previous study (RDS‐test, McGill Vision Research,mcgill.ca/mvr).^15^ The second test was the four circles stereogram (4‐C test, McGill Vision Research, www.mcgill.ca/mvr).

For the RDS‐test, the stimuli were spatially bandpass (peak spatial frequency 2.5 cycles/degree) random dots (average luminance 48 cd/m^2^) on a uniform grey background (luminance 34 cd/m^2^). As mentioned above, the dots seen by the two eyes were offset to give the perception of a floating disk with a missing sector (‘Pac‐Man’ shape). The stimulus subtended an angle of 9° at the 90 cm viewing distance, which was maintained with a head/chin rest. Initial disparity was set at 160 sec arc. Participants reported the perceived position of the sector in depth (up, down, left, right) by selecting a key on a keyboard in a four alternative forced choice procedure without feedback. A staircase method was employed to control the disparity presented for each trial. One staircase was 1‐up‐1‐down and another staircase was 2‐up‐1‐down. Initially, the staircases were in ratio steps of 2, but after the first reversal they were in ratio steps of √2. Each staircase terminated after 70 trials or 9 reversals, whichever occurred first. The data were fitted by a cumulative normal psychometric function. Thresholds were calculated as the disparity required to achieve a 62.5% correct performance level.^15^ A study was carried out to assess the methodology underlying this digital stereoacuity measurement in groups with normal and abnormal binocularity.^15^ The repeatability and expected measurement error were specifically addressed. When assessing test‐retest reliability, an intraclass correlation coefficient of 0.77 was found from the group with normal stereo vision. The mean standard error of the log10‐transformed stereo thresholds was 0.07 in the normal group. In linear terms, this is an error of around 17%.

For the 4‐C test, four 1° diameter circles were presented 2° to the right, left, up and down relative to the centre of the monitor screen. The circles were spatially bandpass, rendered using a fourth‐order derivative of Gaussian function (after Wilkinson et al.^16^). Their peak spatial frequency was 2.5 c/deg. A disparity was applied to one of the circles. This disparity was selected from one of a pair of interleaved staircases, as in the random dot test above. The disparity was applied equally to the right and left eye targets. Unlike the random dot test, the disparity applied to the target circle could be either crossed (right eye target displaced to left side of participant, vice versa for left eye target) or uncrossed (with translations in the opposite direction). After the disparity was calculated, magnification was applied to the stimulus for one eye. The computer‐induced target magnification modified the spacing, size and shape of the targets. Magnification could be either overall or meridional, and could be applied to the right or left eye's targets. The orientation of magnification matched that of a meridional magnifying lens placed in front of an eye e.g., R + 1.0% × 135 corresponded to 1% magnification along the 45° meridian, when looking at a participant's eye, or along the 135° meridian on the screen i.e., left‐upwards to right‐downwards.

All participants underwent training where the tests were explained, and data for one complete run at each test was performed with the habitual correction and subsequently discarded.

### Experiment 1 – comparison between tests

Stereoacuity measures were taken using both the RDS and 4‐C tests under a range of anisometropia and aniseikonia conditions. Data for the conditions described below were collected for each test.

To induce anisometropia, trial lenses were placed in a Keeler Halberg trial clip (keeler.co.uk) in front of the spectacles or at the front of the trial frame. There were six trial lens powers and cylinder axis combinations, placed in front of the right eye. These included no lens (zero or baseline), +2 D and −2 D spheres and +2 D cylinders at 45°, 90° and 180° axes. The trial lens order for each participant was randomised based on the random number generator in Excel (microsoft.com). Three measures were taken for each participant/ power/ axis combination. These were converted into log_10_ seconds of arc, and the mean and standard deviation determined.

To induce aniseikonia, magnifying size lenses^11^ were placed in the front of the right cell of the trial frame. The magnifications were zero, 6% overall and 6% at 45°, 90° and 180° axes. The testing order of magnification lenses for each participant was randomly generated. Three measures were taken for each participant/size lens/axis combination, converted into log seconds of arc, and the mean and standard deviation determined.

### Experiment 2 – effect of peripheral screen masking on the comparison between differential magnifications produced optically by lenses and digitally on the screen – pilot study

Two participants took part in this experiment. For the 4‐C test, the same magnifications as in Experiment 1, except for 6% × 45, were simulated on the screen. Lens and screen effects were then opposed by 6% lens‐induced magnification for the right eye, combined with screen‐simulations of either 6% left eye magnification or 6% right eye minification. Six measures were taken for the zero condition, and three to six measures were taken for the other combinations. Because of concerns about whether the computer surrounds might affect the results, testing was repeated both without and with a peripheral field mask. The mask consisted of a matte black cardboard, 70 cm long hollow frustum between the participant and the screen, with openings 200 and 100 mm diameters so that the former was as close to the eyes as possible, and the latter corresponded to approximately 8° diameter field of view.

### Experiment 3 ‐ comparison between differential magnifications produced optically by lenses and digitally on the screen

Based on the results from Experiment 2 (see Results), masking was used in this experiment and five additional participants were tested under the conditions described for Experiment 2.

### Statistical analysis

For Experiments 1 and 3, the outcomes of the Shapiro‐Wilks test were that only one of 20 data sets and three of 13 data sets, respectively, were not normally distributed; on this basis, parametric statistics were used.

For Experiment 1, repeated measures analyses of variance were applied, with inter‐subject variables for induced anisometropia being test (RDS, 4‐C) and lens condition (zero, +2 D and −2 D spheres and +2 D cylinders at 45°, 90° and 180° axes) and inter‐subject variables for induced aniseikonia being test (RDS, 4‐C) and magnification condition (zero, 6% at 180°, 45° and 90° axes and 6% overall). The Holm‐Bonferroni test was used in *post*‐*hoc* analysis of meridional pair‐wise comparisons.

For Experiment 2, unpaired t‐tests were applied to compare conditions within individuals.

For Experiment 3, two repeated measures analysis of variance were applied: inter‐subject variables for induced aniseikonia being test condition (lens, screen) and magnification condition (6% at 90° and 180° axes and 6% overall); inter‐subject variable of compensating condition (baseline, left eye screen compensation 6% at 90° and 180° axes and 6% overall, and right eye screen compensation 6% at 90 and 180° axes and 6% overall). For the first analysis, the Holm‐Bonferroni test was used in *post*‐*hoc* analysis of meridional pair‐wise comparisons. For the second, a Bonferroni correction of 7 was applied in *post*‐*hoc* analysis of pair‐wise comparisons.

## RESULTS

### Experiment 1 – comparison of random dot stereogram and 4‐C tests

**FIGURE 1:**
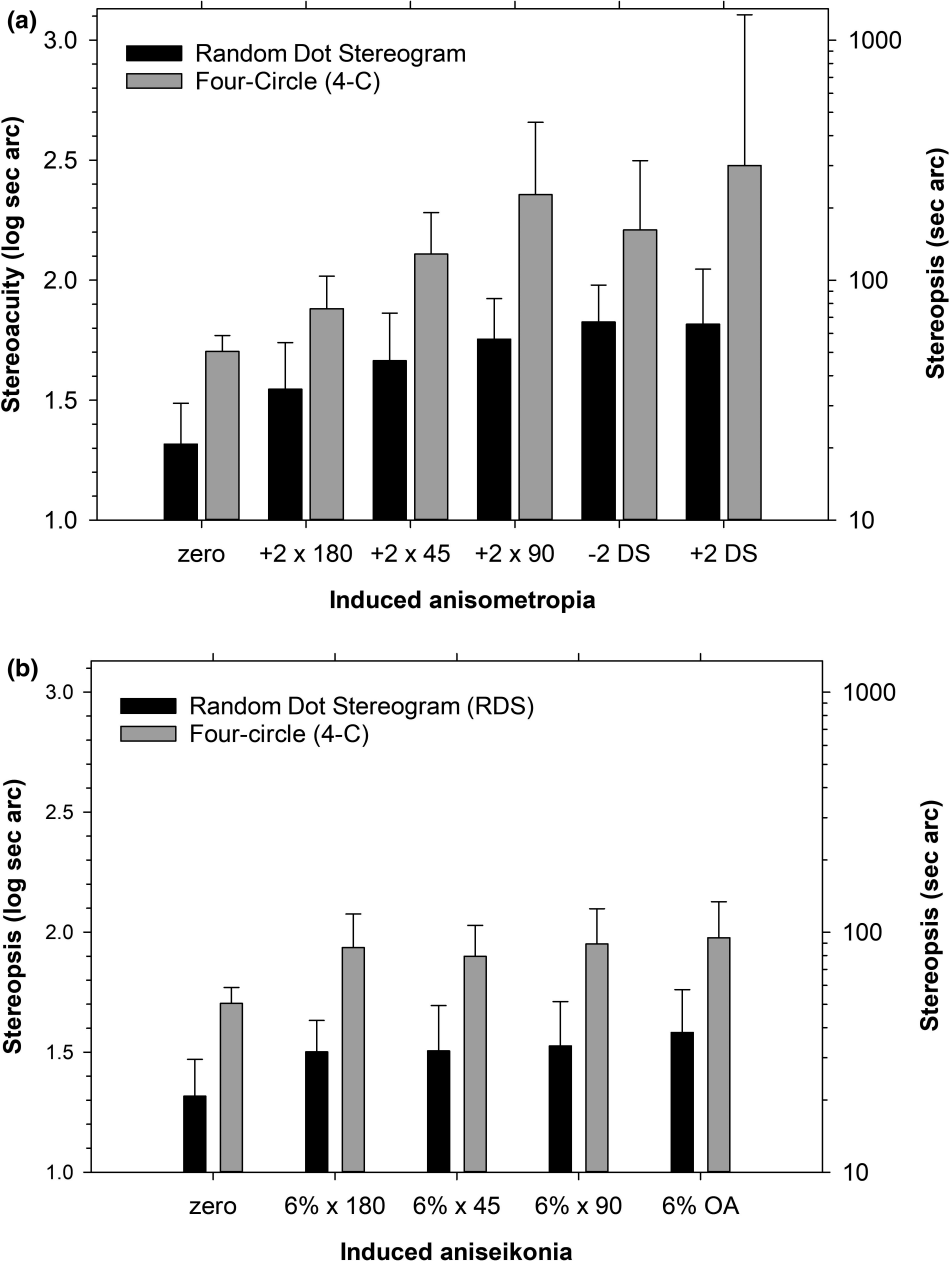
Stereoacuity for random‐dot stereogram and four circle tests in Experiment 1. (a) Results under conditions of induced anisometropia, with powered lenses placed in front of right eyes. (b) Results under conditions of induced aniseikonia, with magnifying lenses placed in front of right eyes. Data are mean ± standard deviations. OA, overall aniseikonia

At baseline, the 4‐C test had higher thresholds by 0.39 ± 0.17 log units (in linear units, mean values were 51 sec. arc for 4‐C and 21 sec. arc for RDS). For induced anisometropia (Figure 1a), test and lens conditions were significant factors (both *p* < 0.001) but their interaction was not significant (*p* = 0.11). The mean difference between tests was 0.47 ± 0.21 log units (3.0 times) across lens conditions. For induced aniseikonia (Figure 1b), test and magnification were significant factors (both *p* < 0.001) but their interaction was not significant (*p* = 0.90). The mean difference between tests was 0.41 ± 0.17 log units (2.6 times) across magnifications.

The order of effects of lens conditions and magnifications were in the direction expected.^11^ Significant effects for anisometropia were 0 < ×180 < all other conditions. Significant effects for aniseikonia were 0 < ×180 <×45, ×90, OA; ×45 < OA (where OA = overall aniseikonia). The important finding is that the effects of anisometropia and aniseikonia were similar for the two tests, i.e., in this respect, the tests are interchangeable.

### Experiment 2 ‐ effect of peripheral screen masking on the comparison of differential magnifications produced optically by lenses and digitally on the computer screen – pilot study

**FIGURE 2:**
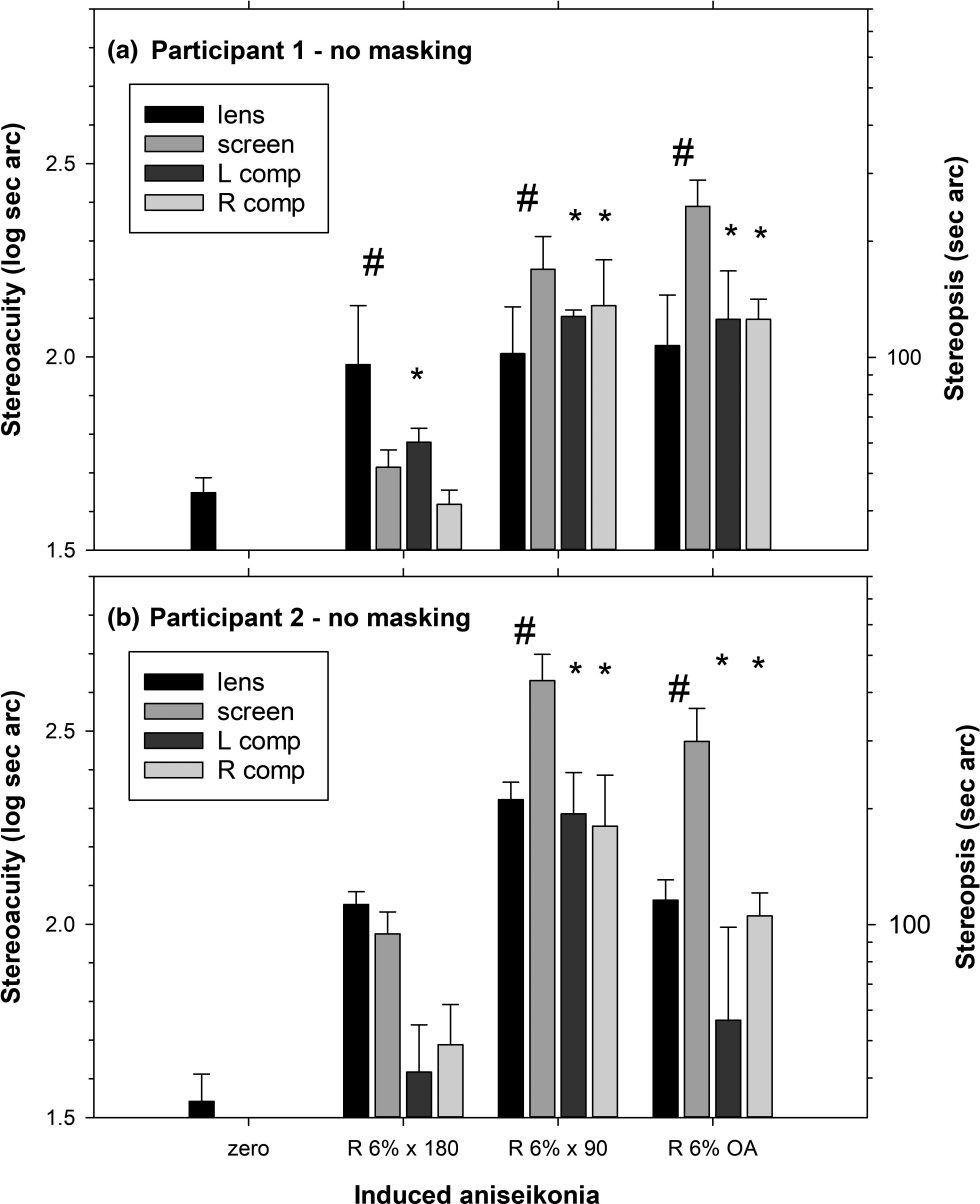
Stereoacuity for four‐circle test for the no masking condition in Experiment 2. (a) Participant 1. (b) Participant 2. Data are means and standard deviations of 3–6 runs. Lens: 6% magnifying lens placed in front of right eyes; screen: 6% screen magnification simulation in front of right eyes; L comp: 6% magnifying lenses placed in front of right eyes and 6% screen magnification for left eye targets; R comp: 6% magnifying lenses placed in front of right eyes and 6% minifying screen simulation of right eye targets. # indicates that lens and screen simulation are significantly different, * indicates that compensations are significantly different from zero condition. OA, overall

**FIGURE 3:**
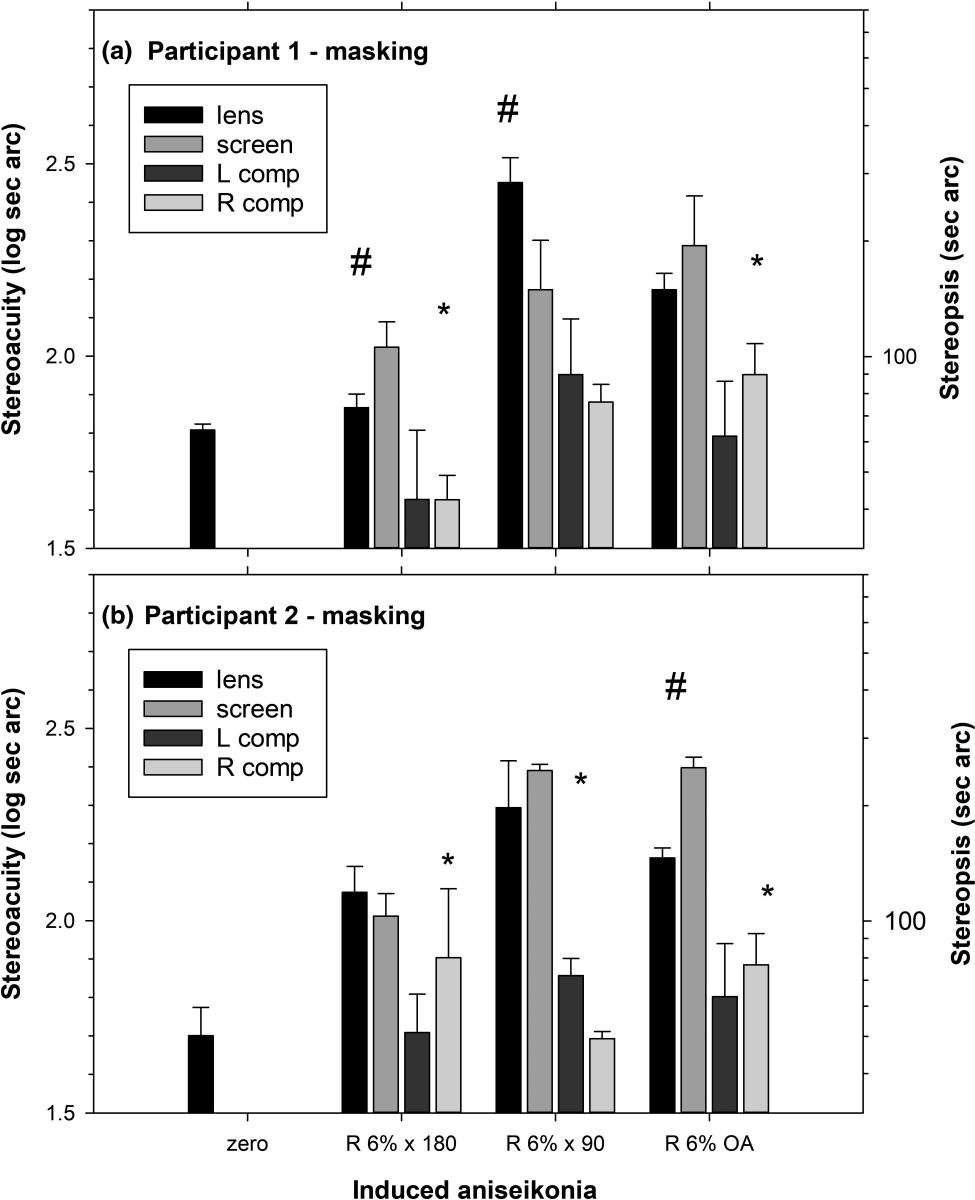
Stereoacuity for four‐circle test for the peripheral field masking condition in Experiment 2. Other details are as for Figure 2

Figures 2 and 3 show results for two participants. For the no masking condition (Figure 2), lens‐ and screen‐induced aniseikonia gave significantly different effects for 5/6 cases (magnifications across two participants), and the compensation conditions gave significantly higher thresholds than baseline in 9/12 cases, indicating incomplete compensation. For the peripheral field masking condition (Figure 3), lens‐ and screen‐induced aniseikonia gave significantly different effects for 3/6 cases and the compensation condition gave significantly different thresholds than baseline in 5/12 cases.

### Experiment 3 ‐ comparison between differential magnifications produced optically by lenses and digitally on the computer screen

For the no masking condition, the two participants in the pilot experiment showed considerable differences in lens‐ vs digitally‐induced aniseikonia, and in most cases compensation of lens‐induced aniseikonia by screen‐induced magnification was only partial. The number of cases in which this occurred were reduced considerably by peripheral masking; therefore, masking was used in Experiment 3.

**FIGURE 4:**
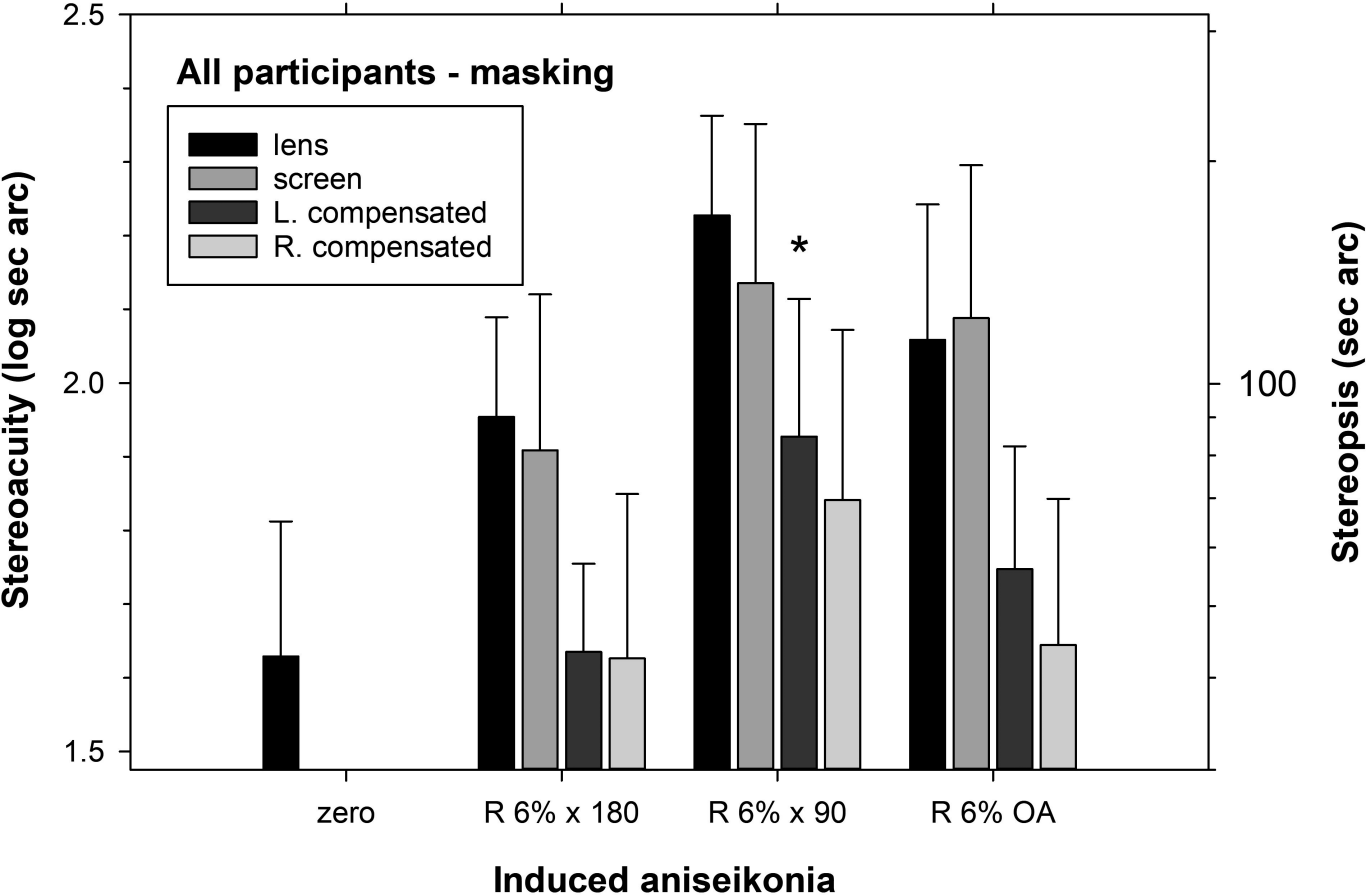
Stereoacuity for four‐circle test for seven participants with peripheral field masking in Experiment 3. Participants 1 and 2 are included. * indicates that compensation is significantly different from zero condition. Other details are as for Figure 2

Figure 4 shows stereoacuity results of the 4‐C test for seven participants with masking. There was no significant difference between thresholds for lens‐ and screen‐induced aniseikonia (F_1, 8_ = 31.47, *p* = 0.27). Unlike Experiment 1, there was a considerable influence of meridian on thresholds (*F*_2, 12_ = 1.96, *p* = 0.001) with the ×90 meridian having greater thresholds than ×180 by 0.3 log unit (*p* = 0.01) and overall magnification by 0.2 log unit (*p* = 0.03). For the compensation conditions, there was a significant effect on threshold (*F*_6, 36_ = 3.89, *p* = 0.004), but only the L compensated ×90 condition was different from the baseline (0.3 log unit, *p* = 0.04). There was excellent compensation for the x 180 and overall magnification conditions.

**FIGURE 5:**
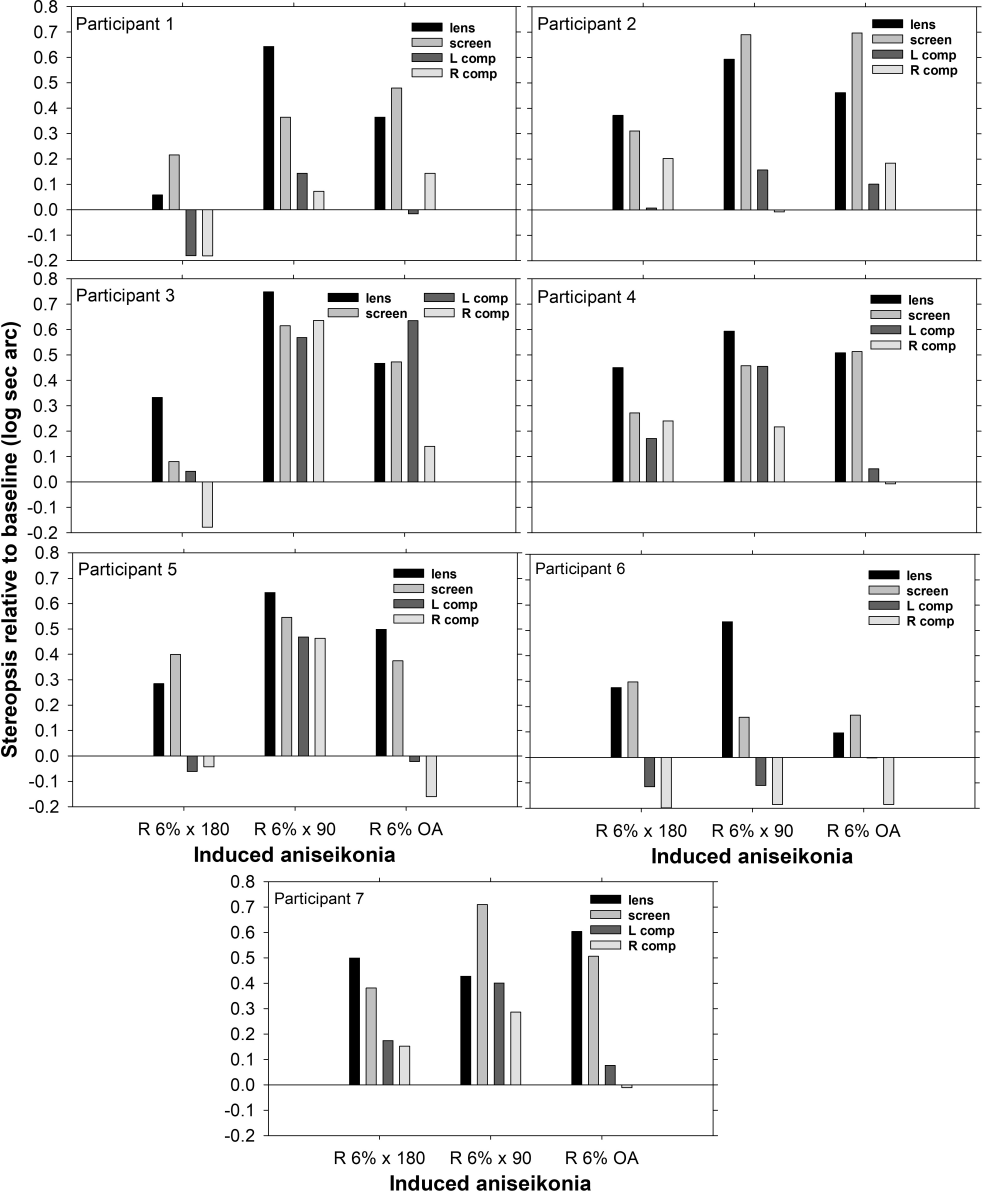
Stereoacuity, relative to baseline results, for four‐circle test for individual participants with peripheral field masking in Experiment 3. Data are the averages of three runs per test condition

Figure 5 shows the stereoacuity differences between the relevant baseline and magnification conditions for individual participants. Two participants had broadly the “classical” pattern of aniseikonia having similar effects on stereoacuity for all meridional conditions^10^ (Participants 4 and 7), but five had greater effects ×90 of which four had the pattern of ×90 > OA > ×180. Although there were considerable inter‐individual differences, there was no “outlier” who distorted the mean results. Three participants showed good compensation for all lens conditions (Participants 1, 2 and 6), while the other four had poor compensation for the ×90 condition. While the mean compensations were similar at 0.32 ± 0.08 (×180), 0.30 ± 0.23 (×90) and 0.32 ± 0.19 (OA), it must be appreciated that ×90 required greater full compensation than the other lens conditions.

## DISCUSSION

To understand better the impact that aniseikonia has on stereopsis and how best to measure aniseikonia, we used a stereopsis test involving four circles (4‐C) whose spatial properties could be modified on the screen to simulate the magnification effects of lenses. The spatial scaling changes were designed to replicate the effect of optical aniseikonia, except that effects were localised to the circles themselves, and head and eye movements were not taken into account.

Comparison of the RDS and 4‐C tests for a range of lens‐induced anisometropic and aniseikonic conditions showed that while stereoacuity thresholds were about 0.4 log units higher with the 4‐C test, the two tests were interchangeable in that thresholds were similarly affected by blur and magnification. We believe that this difference reflects the difficulty of the 4‐C task, and that there is additional induced disparity leading to a confusing depth decision; this is further explored later.

When the 4‐C test was used with lens‐induced magnification and screen simulations of magnification in Experiment 4 with screen periphery masking, results were similar for the lens‐induced and screen simulations. However, here the effect of ×90 magnification was much greater than for ×180 and overall magnifications. Screen‐based compensations for the lens‐induced effects were successful in returning results to baseline levels for ×180 and overall conditions, but this was only partially successful for ×90 magnification, and varied between participants. In short, we were not able to fully replicate the effects of lens‐induced aniseikonia on stereopsis simulations by replicating the physical effects on the screen. One possible contributor to the difference between lens‐induced and screen‐based effects is that for the former, the whole field of view, which includes the edges of the monitor and beyond, is affected by magnification, whereas for the latter only the four circles are affected.

It is known from the pioneering work of Ogle^17^ that meridional magnification at axis 90% produces a rotation of the horopter, the so‐called geometric effect, and that this affects the whole visual field (as demonstrated by the globally perceived distortions to the Ames leaf room). What this means is that the plane of reference for the stereo judgements was not constant for all the circular stimuli, rendering the judgement of depth more difficult because the reference plane was no longer at zero disparity. Subjects would also be taking this global context into account in the optical condition, and this would render the image‐based compensation less effective. This finding is relevant to digital clinical tests such as the Awaya Aniseikonia Test and the Aniseikonia Inspector, which measure aniseikonia by a screen‐based manipulation. Unsurprisingly considering the current results, it has been shown that these methods tend to underestimate lens‐induced aniseikonia.^18^, ^19^ What we show here is that this has a meridional dependence as well as subject‐based variability in terms of its implications for stereopsis.

It should be remembered that with lens‐induced aniseikonia, the induced image size difference between the two eyes distorts the entire horopter plane and thus the ability to accurately determine depth. Using the computer display, the reduction in stereoacuity can then be measured. In the case where the size difference is simulated using computer‐generated targets, this occurs only for the four circles (one in each quadrant) and here it is unlikely that the entire horopter plane is distorted. This could explain the need for the peripheral field mask.

These differences in lens‐induced aniseikonia and display‐induced interocular target size difference would also affect the outcomes of the experiment aimed to determine if the lens‐induced aniseikonia could be countered using computer‐generated targets. This was achieved reasonably well for overall and vertical, but not for horizontal aniseikonia. It is possible that for the horizontal meridian, the mask could not adequately eliminate all of the peripheral cues, particularly where vergence and accommodation links would likely have greater roles. We speculate that the peripheral mask did not eliminate all distortion to the peripheral reference plane because the mask itself was visible. It is virtually impossible to provide a mask that is itself invisible at its edges. The visible mask edges would have been subject to lens distortion, and therefore could still have affected the reference plane from which the central stereo judgement was made.

We have shown that it is possible, at least to some extent, to negate the effects on stereopsis of lens‐induced aniseikonia by directly altering the size of targets presented to each eye on a computer screen. This has possible applications to the virtual reality industry, where individuals with poor stereoacuity have reduced performance, and in the case where some of this may be due to aniseikonia, it might be possible to compensate for this using the display.

## CONCLUSION

The effects of lens‐induced aniseikonia on stereopsis cannot always be successfully simulated by a screen‐based method. The ability to neutralise a person's refractive aniseikonia using a computer screen‐based method, which is the basis of digital clinical measurement, was reasonably successful for overall and ×180 meridional aniseikonia but not for ×90 aniseikonia. We hypothesise that optical distortions that extend well beyond the display are considered by the visual system in more global processing when stereopsis is measured. This has implications for x 90 meridional aniseikonia, and the calculation of compensating size lens corrections need to take this into account.

## CONFLICTS OF INTEREST

Robert Hess and Alex Baldwin are inventors on a PCT Patent Application (CA2020050051) titled “System and Method for Digital Measurement of Stereo Vision”, filed 17 January 2020, which has been commercially licensed by McGill University.

## AUTHOR CONTRIBUTION

**David Atchison:** Conceptualization (equal); Investigation (equal); Methodology (equal); Project administration (equal); Supervision (equal); Writing – original draft (equal). **Thien Nguyen:** Data curation (equal); Formal analysis (equal); Investigation (equal); Methodology (equal). **Katrina L Schmid:** Conceptualization (equal); Methodology (equal); Resources (equal); Writing – review & editing (equal). **Archayeeta Rakshit:** Investigation (equal); Writing – review & editing (equal). **Alexander Baldwin:** Resources (equal); Software (equal); Writing – review & editing (equal). **Robert F Hess:** Resources (equal); Writing – review & editing (equal).

## References

[1] Wu KT , Lee GS . Influences of monocular and binocular vision on postural stability. J Vestib Res 2015;25:15–21. 25882473 10.3233/VES-150540

[2] Johansson J , Pansell T , Ygge J , Seimyr G . Monocular and binocular reading performance in subjects with normal binocular vision. Clin Exp Optom 2014;97:341–8. 24612111 10.1111/cxo.12137

[3] McKnight AJ , Shinar D , Hilburn B . The visual and driving performance of monocular and binocular heavy‐duty truck drivers. Accid Anal Prev 1991;23:225–37. 1883464 10.1016/0001-4575(91)90002-m

[4] Kelly KR , Jost RM , Dao L , et al. Binocular iPad game vs gatching for treatment of amblyopia in children: a randomized clinical trial. JAMA Ophthalmol 2016;134:1402–8. 27832248 10.1001/jamaophthalmol.2016.4224 PMC6054712

[5] Grant S , Moseley MJ . Amblyopia and real‐world visuomotor tasks. Strabismus 2011;19:119–28. 21870915 10.3109/09273972.2011.600423

[6] Adrian J , Le Brun J , Miller NR , et al. Implications of monocular vision for racing drivers. PLoS One 2019;14:e0226308. 10.1371/journal.pone.0226308 31841526 PMC6913915

[7] Webber AL , Wood JM , Thompson B . Fine motor skills of children with amblyopia improve following binocular treatment. Invest Ophthalmol Vis Sci 2016;57:4713–20. 27607417 10.1167/iovs.16-19797

[8] Loftus A , Servos P , Goodale MA , Mendarozqueta N , Mon‐Williams M . When two eyes are better than one in prehension: monocular viewing and end‐point variance. Exp Brain Res 2004;158:317–27. 15164152 10.1007/s00221-004-1905-2

[9] Heinen T , Vinken PM . Monocular and binocular vision in the performance of a complex skill. J Sports Sci Med 2011;10:520–7. 24150627 PMC3737810

[10] Ostadimoghaddam H , Fotouhi A , Hashemi H , et al. Prevalence of the refractive errors by age and gender: the Mashhad eye study of Iran. Clin Exp Ophthalmol 2011;39:743–51. 21631683 10.1111/j.1442-9071.2011.02584.x

[11] Atchison DA , Lee J , Lu J , et al. Effects of simulated anisometropia and aniseikonia on stereopsis. Ophthalmic Physiol Opt 2020;40:323–32. 32128857 10.1111/opo.12680

[12] Atchison DA , Schmid KL , Haley EC , et al. Effects of binocularly‐induced blur and aniseikonia on stereopsis. Ophthalmic Physiol Opt 2020;40:660–8. 32776575 10.1111/opo.12724

[13] Smith J , Gao T , Collins A , et al. Aniseikonia and anisometropia: implications for suppression and amblyopia. Clin Exp Optom 2019;102:556–65. 30791133 10.1111/cxo.12881

[14] de Wit GC . Evaluation of a new direct‐comparison aniseikonia test. Binoc Vision Strab Quart 2003;18:87–94. 12765541

[15] Tittes J , Baldwin AS , Hess RF , et al. Assessment of stereovision with digital testing in adults and children with normal and impaired binocularity. Vision Res 2019;164:69–82. 31377344 10.1016/j.visres.2019.07.006

[16] Wilkinson F , Wilson HR , Habak C . Detection and recognition of radial frequency patterns. Vision Res 1998;38:3555–68. 9893789 10.1016/s0042-6989(98)00039-x

[17] Ogle KN . Researches in binocular vision. Philadelphia & London: W. B. Saunders. 1950.

[18] Antona B , Barra F , Barrio A , Gonzalez E , Sanchez I . Validity and repeatability of a new test for aniseikonia. Invest Ophthalmol Vis Sci 2007;48:58–62. 17197516 10.1167/iovs.05-0575

[19] Rutstein RP , Corliss DA , Fullard RJ . Comparison of aniseikonia as measured by the aniseikonia inspector and the space eikonometer. Optom Vis Sci 2006;83:836–8. 17106411 10.1097/01.opx.0000238722.34167.cc

